# Outbreak Investigation, Isolation, and Molecular Characterization of Lumpy Skin Disease Virus in Cattle from North West Oromia Region, Ethiopia

**DOI:** 10.1155/2024/6038724

**Published:** 2024-08-16

**Authors:** Workisa Chala, Kasaye Adamu, Hawa Mohammed, Getaw Deresse, Shimelis Tesfaye, Esayas Gelaye

**Affiliations:** ^1^ National Veterinary Institute, P.O. Box 19, Bishoftu, Ethiopia; ^2^ College of Veterinary Medicine and Agriculture Addis Ababa University, P.O. Box 34, Bishoftu, Ethiopia; ^3^ Food and Agriculture Organization of the United Nations Sub-Regional Office for Eastern Africa, P.O. Box 5536, Addis Ababa, Ethiopia

## Abstract

Lumpy skin disease (LSD) is an economically significant viral disease because of its high morbidity and high production loss. Vaccination of cattle using LSD vaccines is a more effective disease preventive and control strategy in endemic countries such as Ethiopia. Despite high vaccination coverage, there is an increasing number of field reports of the disease outbreaks. Thus, an observational study was designed to investigate disease, characterize the disease-causing agent, and isolate the virus from a local isolate for future vaccine development. Wera Jarso and Amuru districts in North West Oromia were chosen based on outbreak occurrence. For this study skin, 13 pooled biopsy samples were collected from affected cattle. In this outbreak investigation, the morbidity rate was 6.50%, the mortality rate was 0.50%, and the case fatality rate was 7.77%. The virus was isolated from all skin samples on both lamb testis and lamb kidney primary cells and confirmed to be LSDV using conventional and real-time PCR genotyping. Therefore, after each suspected LSD outbreak, a molecular test should be carried out to confirm the cause of the disease, targeting the previously suggested RPO30 or GPCR genes. Further studies targeting more regions and outbreaks, including full genome sequencing to check for genetic differences between the field viruses and vaccine strains, are recommended.

## 1. Introduction

Ethiopia has Africa's largest livestock population. This livestock sector has made a significant contribution to the country's economy. Livestock contributes to the national economy, particularly in terms of foreign currency earnings, through an exploration of a live animal, meat, and skin and hides. Livestock production is still important and serves as a valuable resource for resource limited smallholder farmers and pastoralists by providing milk, meat, skin, manure, and traction force [1]. Lumpy skin disease has drastically reduced the health and productivity of Ethiopian cattle, resulting in economic losses [2].

Lumpy skin disease (LSD) is an acute to subacute cattle disease characterized by skin nodules, pox lesions in ocular, nasal, and oral mucous membranes, as well as on the surface of internal organs, skin edema, fever, lymphadenitis, and, in some cases, mortality, which causes significant economic losses, particularly in the hide industry [3]. LSD is caused by a virus classified in the *Capripoxvirus* of the family *Poxviridae*. The virus strains that cause sheeppox and goatpox cannot be differentiated serologically; however, molecular techniques can be utilized to genotype them [4].

Vaccination, movement control, and deliberate slaughter of infected and in-contact animals are among the measures for disease control but slaughtering of infected and in-contact animals does not appear to be economically feasible in resource-poor countries where the disease is endemic. Vaccination on the other hand is widely accepted as the most manageable and realistic approach to disease control in endemic and resource-poor countries [5]. In Ethiopia, a live attenuated vaccine based on the sheeppox virus Kenya O-180 vaccine strain (referred to as KS-1, which is an LSD virus) of Kenyan origin has been used to control LSD [6]. Vaccination helps to directly protect vaccinated animals, reduce the severity of the disease by reducing all or some of its symptoms, or it may reduce pathogen transmission by lowering susceptibility and infectiousness, and thus indirectly reduces the risk of infection for other vaccinated and unvaccinated individuals [7]. Immunization failure can occur as a result of insufficient vaccine coverage or factors related to the host, vaccine, or vaccination quality due to vaccine handling, storage, reconstitution, or administration [8].

Several countries including Ethiopia have reported LSD vaccine failure [9–12]. Even though the vaccination coverage is high in Ethiopia, there is an annual LSD outbreak; as a result, there is a high motivation for outbreak investigation to determine LSD vaccine failure [13]. Molla et al. found that Ethiopian LSD vaccine has low efficacy and recommended either improving the vaccine's quality or developing alternative vaccine with higher efficacy [14]. Capripox diseases are highly significant in Ethiopia due to the insufficient vaccination coverage [15]. There is also a dearth of understanding of the partial protection provided by vaccination, and there have been few investigations on the circulating strain of lumpy skin disease virus (LSDV) in Ethiopia. For better control of capripoxviruses (CaPVs) in Ethiopia, the development of a new more efficient vaccine that matches with the local outbreak isolate is recommended [15]. The vaccine failure could also be attributed to the vaccinal strain's lack of cross-protection against the circulating field strain. Therefore, in order to develop an effective and potent vaccine, the circulating field LSD viruses are characterized using genome sequencing. Therefore, the objectives of this study were to investigate outbreaks from different areas, to isolate the field LSDV using cell cultures, and to characterize the isolated LSDV using molecular techniques.

## 2. Materials and Methods

### 2.1. Study Area

**Figure 1:**
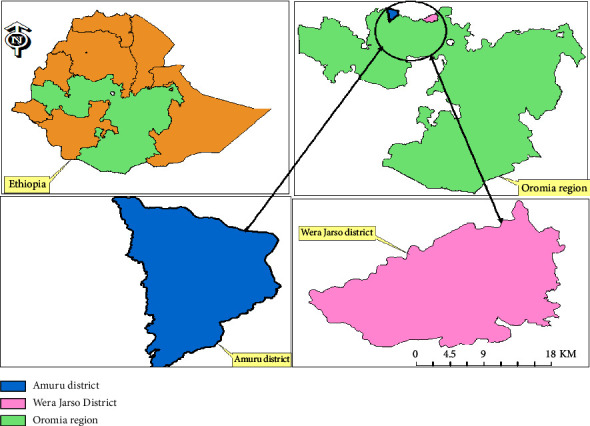
Map of study areas created by ArcGIS (ArcMap 10.2).

The study was conducted from October 2019 to April 2020 in two selected areas of North West Oromia regional state of Ethiopia, based on the LSD outbreak report. Samples were collected from North Shoa, Wera Jarso district, and Horo Guduru Wolega, Amuru district. Wera Jarso is located in the North Shoa zone of the Oromia regional state (Figure 1). The district is located at 185 km from Addis Ababa on the main road connecting Addis Ababa to Gondar at 9050′N 38015′ E. Amuru district is one of the districts of the Horo Guduru Wolega zone which is located 392 km northwest of Addis Ababa. This district is located at 09°59′23′N 37°01′44′E and has an elevation of 960 meters. The laboratory investigation was carried out at the National Veterinary Institute (NVI) in Bishoftu, 47 km southeast of Addis Ababa.

### 2.2. Study Animals

Cattles with clinical signs of pox-like skin lesion were chosen for this study. All cattle used in the study were local breed of both sexes, with no exceptions made for the cattle age. An outbreak investigation was carried out to collect primary data from veterinarians and owners using semistructured questions about the total number of cattle, a diseased animal, and animals died and the clinical findings.

### 2.3. Sample Collection

The purposive sampling strategy was used to collect samples from the outbreak area. Sick animals were examined physically before being collected, and nodular skin samples were collected aseptically from clinically sick animals displaying typical clinical signs of LSD following the standard sample collection procedure. There were 13 pooled specimens collected, 9 from Wera Jarso and 4 from Amuru. Tissue samples were placed in the sterile universal bottle containing virus transport media, labeled, and transported to the National Veterinary Institute maintaining the cold-chain system. The tissue samples were stored at −20°C until processed.

### 2.4. Laboratory Investigation

#### 2.4.1. Sample Processing

The skin biopsy samples were thawed at room temperature and washed three times using sterile phosphate buffer saline (PBS, pH 7.2) in the Biosafety Cabinet Class II. Using sterile scissors and forceps, the tissue samples were minced. Approximately one gram of the sample was ground in a sterile pestle and mortar with 9 ml of sterile PBS containing 0.1% antibiotic. The tissue suspension was centrifuged at 600 × g for 15 minutes, and the supernatant was collected, labeled, and stored at −20°C until use.

#### 2.4.2. Primary Cell Culture Preparation

To obtain lambs for the preparation of lamb testis and lamb kidney primary cells, four pregnant ewes with varying pregnancy levels were purchased from the local market. Newly born lambs were slaughtered humanely when they were 7–10 days old; no injections were given because this procedure may affect the viability of the primary kidney cells. The four ewes were transferred to the institute's animal farm. After removal of the skin, the kidneys and testis were aseptically taken to the biosafety cabinet. The cells were then seeded in 75 cm^2^ tissue culture flasks with Glasgow minimum essential medium (GMEM) containing 10% fetal calf serum and antibiotics at 10^6^ cells/ml. The medium in each flask was changed every 24 to 48 hours depending on cell growth.

#### 2.4.3. Virus Isolation

Each sample was subjected to conventional PCR to determine the presence of an infectious virus. To isolate the virus, the clarified supernatant was inoculated onto the lamb kidney and lamb testis primary cells using the method described by Balinsky et al. [16]. Glasgow eagle minimal essential medium (GMEM) supplemented with 10% calf serum (Gibco) and 10% tryptose phosphate broth (TPB) was used to propagate the primary cells. Each primary cell was grown in a 75 cm^2^ tissue culture flask and was incubated with 5% CO_2_ at 37°C until it formed a confluent monolayer. The medium was then removed aseptically, and the monolayer washed three times in biosafety cabinet level II with sterile warm PBS (pH 7.2). One ml of clarified supernatant was inoculated onto the confluent monolayer and incubated at 37°C for 1 hour before adding 10 ml of GMEM containing antibiotics and 2% fetal calf serum and placing it in an incubator. Monolayer cells were monitored daily using an inverted microscope for evidence of a virus-induced cytopathic effect (CPE) for 7–10 days postinoculation. Infected cells displayed a characteristic CPE that included retraction of the cell membrane from surrounding cells, followed by rounding and aggregation. After observing 80% cytopathic effect (CPE), virus-inoculated flasks were harvested and frozen overnight at −20°C. The harvested cell culture was freeze-thawed three times at room temperature to release the virus particles. Finally, virus suspensions were stored at −20°C until viral DNA detection was performed.

#### 2.4.4. DNA Extraction

Viral DNA was extracted using the DNeasy® blood and tissue kit (QIAGEN, Germany) following the manufacturer's instruction. DNA was extracted from 13 outbreak investigation specimens and 6 samples inoculated on primary cells for virus isolation. DNA was eluted in 200 *μ*l of elution buffer.

#### 2.4.5. Conventional PCR and Gel Electrophoresis

The LSD virus was detected using the polymerase chain reaction (PCR) with capripoxvirus specific primers: SpGpRNAPol-For: 5′-TCTATGTCTTGATATGTGGTGGATAG-3′ and SpGpRNAPol-Rev: 5′-AGTGATTAGGTGGTGTATTATTTTCC-3′, previously designed by Lamien et al. [17]. Master mix was prepared in a total volume of 20 *μ*l for one reaction, which contain 10 *μ*l super mix, 2 *μ*l forward primer, 2 *μ*l reverse primer, 3 *μ*l RNase free water, and 3 *μ*l template DNA. The PCR tube was transferred into a thermal cycler, and amplification was conducted using the following program: initial denaturation at 95°C for 5 minutes followed by 40 cycles at 95°C/30 sec, annealing at 50°C for 30sec, and extension at 72°C for 30 sec, and a final extension at 72°C for 7 minutes. Aliquots of 5 *μ*l PCR products were analyzed using a 3% agarose gel stained with Gel-Red (Biotium, Inc.) at 100 volts for 1 hour.

Amplified products were analyzed using a Gene-Ruler™ 100bp DNA ladder (Fermentas, Germany) as a molecular marker on a 3% agarose gel prepared according to the manufacturer's instructions. Ten *μ*l of the PCR product was mixed with 4 *μ*l of loading buffer and loaded into prepared gel which was then run at 100 volts for about 60 minutes in the electrophoresis apparatus until the DNA migrated. The PCR products were visualized using a UV transilluminator, and positive results were confirmed by the size of the bands.

#### 2.4.6. Real-Time PCR

The samples were genotyped using the previously designed molecular method for CaPVs [18], which uses unlabeled snapback primers, dsDNA intercalating dye, and high-resolution melting (HRM) analysis. The PCR assay distinguishes CaPVs based on the fluorescent melting peaks targeting the CaPV RPO30 gene, and it is useful for detecting and genotyping field isolates of CaPVs [18]. Real-time PCR was carried out using the amplification primers and PCR protocol as described by Gelaye et al. [18]. The primers used are snapback forward primer: 5′-GGTGTAGTACGTATAAGATTATCGTATAGAAACAAGCCTTTA-3′ and reverse primer: 5′-AATTTCTTTCTCTGTTCCATTTG-3′. The RT-PCR master mix was prepared in a reaction volume of 20 *μ*l containing 4.84 *μ*l of RNase free water, 2 *μ*l of forwarding primer, 0.16 *μ*l of reverse primer, 10 *μ*l of SsoFast Eva-Green® super-mix, and 3 *μ*l template DNA. Using a low-profile Hard-Shell® 96 well PCR plate (Bio-Rad), the PCR reaction was run with an initial denaturation at 95°C for 3 minutes, followed by 40 cycles of denaturation at 95°C for 15 seconds, annealing, and extension at 58°C for 80 seconds. To perform melting curve analysis, the product was then denatured at 95°C held for 1 minute, cooled to 40°C, and heated continuously at 0.5°C for 10 seconds with fluorescence acquisition from 45°C to 85°C. No template and positive GTPV, LSDV, and SPPV controls were included in the real-time PCR analysis. DNA samples were tested in duplicates.

The melting temperatures were analyzed using CFX™ Manager Software version 2.0 (Bio-Rad). The Precision Melt Analysis™ software (Bio-Rad) was used to plot the melting profile of the three genotypes using high-resolution melting (HRM) analysis. Normalized melt curves and differences in curves were acquired by selecting separately pre- and postmelt regions for amplicon [18].

### 2.5. Data Analysis

The collected data during sample collection and laboratory investigation were coded and stored in the Microsoft Office Excel spreadsheet 2010. The data were thoroughly screened, and descriptive statistics were used to summarize the data.

## 3. Results

### 3.1. Observed Clinical Signs

**Figure 2:**
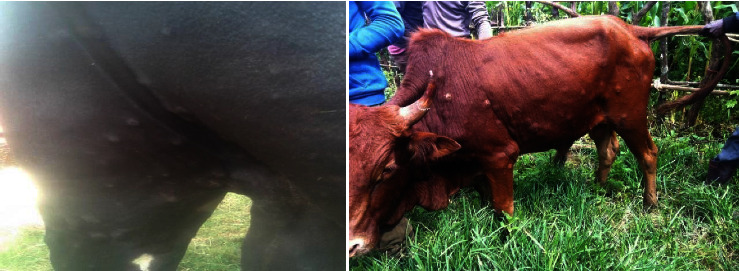
Photo taken during the outbreak where characteristics of LSD with generalized skin nodules covering the entire body.

In this study, the most common clinical signs observed were fever, nodules on the skin, necrotic nodules and deep scab formation, edematous swelling of one or two legs, and enlargement of superficial lymph nodes. Lameness and superficial lymph node enlargement were very prominent. The nodules on the limbs gradually burst and leaving necrotic wounds that were frequently complicated by secondary infection (Figure 2).

From the two districts where outbreak investigations of LSD were conducted, Amuru district had a slightly higher morbidity rate (9.93%) as compared with W/Jarso district (3.73%), whereas the mortality rate is 0.82% and 0.25% for Amuru and W/Jarso districts, respectively. The case fatality rate was almost similar as shown in Table 1.

### 3.2. Virus Isolation

**Figure 3:**
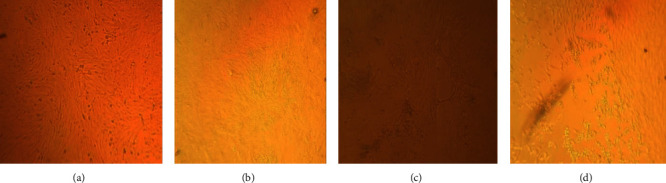
LSDV growth in primary cell cultures, where (a) control lamb kidney cells; (b) control lamb testis cells; (c) lamb kidney cell showing CPE; (d) lamb testis cell showing CPE.

All of the collected and purified skin biopsy samples were inoculated into the lamb testis and lamb kidney primary cells prepared as described above, and the virus culture was passaged three times to achieve a high titer for future use as a challenge virus. Both primary cell cultures used showed the characteristic LSDV cytopathic effect (CPE), with lamb testis cells showing better CPE (Figure 3). The CPEs were characterized by the rounding of single cells, the aggregation of dead cells, and the destruction of the monolayer.

### 3.3. Conventional PCR DNA Amplification

**Figure 4:**
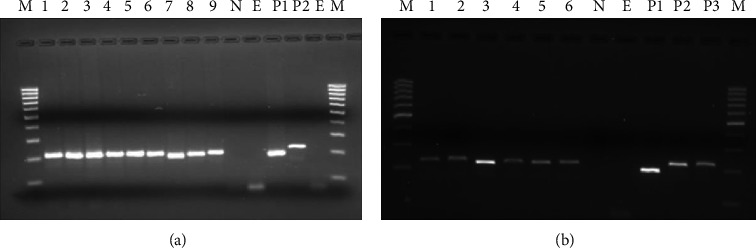
Classical PCR gel images from infected cattle (a) lane M: DNA ladder (100bp Fermentas), lanes 1 to 9: positive field samples, lane N: negative control, lanes P1 and P2: positive controls, and lane E: extraction control. From cell culture, (b) lanes 1 to 6: positive field samples isolated on primary cells and lane N: negative control without template; NE is a negative control for extract, P1 is a positive control for SPPV, P2 is a positive control for LSDV, and P3 is a positive control for GTPV.

The DNA of 13 LSDV isolates and 6 LSDV samples from primary cell lines were amplified using capripoxvirus specific primers. Goatpox virus (GTPV) and LSDV PCR products differed in length by 21 nucleotides produced from sheeppox virus (SPPV) genomes. The positive control amplicon for SPPV was shorter (151 bp) and easily distinguishable at the end of electrophoresis migration of the PCR products on a 3% agarose gel, compared to the GTPV positive control and positive control LSDV amplicons 172 bp (Figure 4).

### 3.4. Real-Time PCR

**Figure 5:**
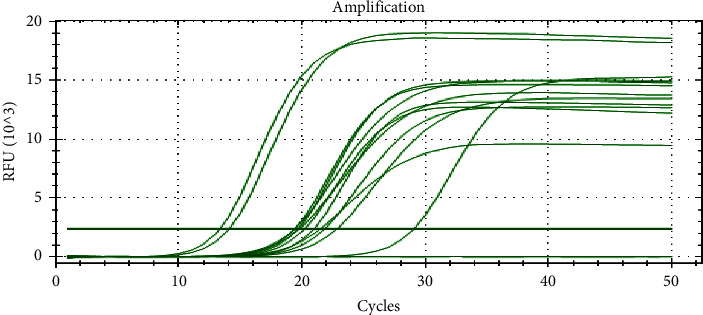
Real-time PCR amplification of LSDV field isolates.

The DNA extracts that revealed 172 bands on agarose gel were again subjected to real-time PCR to distinguish LSDV from GTPV. The samples were genotyped using the previously developed real-time PCR assay [17]. On average, all collected samples started amplification at 19 cycles on average (Figure 5).

**Figure 6:**
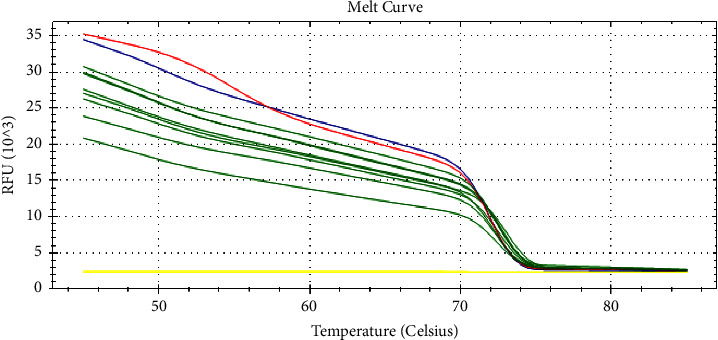
Melting curve profile of field isolates and positive controls.

The real-time peak melting curve revealed that all 13 field-LSDV isolates were genotyped as LSDV since their snapback of melting peaks were at 51°C and the second peaks were at 73.5°C. Known LSDV, GTPV, and SPPV positive samples were included. After fluorescence melting curve analysis, real-time PCR assay detected differences in the melting point temperatures for CaPVs (Figure 6).

**Figure 7:**
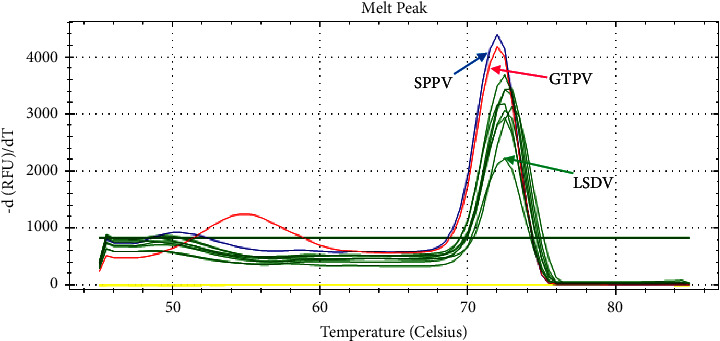
Melting peak of LSDV field isolates and CaPV positive controls.

For genotyping of the tested isolate, a pair of melting temperatures for snapback tail and the full amplicon was recorded as LSDV at 51°C/73.5°C, GTPV at 56°C/72.5°C, and SPPV at 52°C/72.5°C (Figure 7). All tested samples were LSDV.

## 4. Discussion

The study confirmed that the outbreak was caused by LSDV. As a result, using conventional and real-time PCR, all 13 biopsy samples from typical clinical cases studied in the current study were confirmed as positive for LSD. Fever, skin nodules, enlargement of lymph nodes, decreased appetite, lacrimation, salivation, decreased milk production, and death are the clinical signs observed, and these clinical signs have been documented as characteristic clinical features of LSD [3, 16, 19]. The clinical manifestations observed in the current study are also in agreement with those documented by Ayelet, Gari, Alemayehu, and Mesay in different areas of Ethiopia [20–23].

The disease's morbidity rate ranges from 5 to 100%, but less than 5% is usually observed [24]. In the current study, 6.50% (1120/17242) morbidity and 0.50% (87/17242) mortality were recorded, as shown in Table 1. Several studies have reported that the morbidity and mortality rates associated with LSD vary. These findings are lower than reports of 13.5% of the morbidity rate by Brenner [10]. The reported morbidity rate agrees with that reported by Alemayehu [22] reported 6.1%, Regassa [25] reported 7.02%, Gari [21] reported 8.1%, and Hailu [26] reported 7.4% morbidity rate in north-eastern Ethiopia, Mesay [23] reported 8.77% morbidity rate in central Ethiopia, and Tasioudi [27] reported an 8.7% LSD morbidity rate in Greece. The reported morbidity and mortality rates are consistent with the report by Kasim, who reported a 6% morbidity rate and a 0.99% mortality rate in Saudi Arabia [28]. As a result, the morbidity and mortality rates in this study are within the ranges reported in previous studies. Furthermore, morbidity and mortality rates varied significantly between districts, with Amuru district reporting the highest morbidity and mortality rates of 9.93% and 0.82%, respectively.

The locally produced Kenyan sheeppox vaccine has been used to immunize cattle against LSD. The vaccine strain SPPV Kenya O-180 confirmed to be a LSDV [15, 17, 18]. However, the data revealed that LSD caused morbidity and mortality, regardless of vaccination status. A similar phenomenon has previously been reported in Ethiopia [20]. This lack of protection could be attributed to the lack of cross-protection of sheeppox vaccine strain against circulating virulent LSDV field strains. All samples were tested for the presence of LSDV DNA by conventional PCR using a specific primer to amplify the RPO30 gene. The amplified gene fragment size of the LSDV positive control and all the local field isolates had the same size 172 base pair. Genotyping was performed because of the reason that LSD confusion may occur with SPPV, as it is rarely transmitted to cattle, and experimental infection of cattle with SPPV can produce similar lesions to LSD [29]. The results showed that the isolated samples are LSDV with an average melting temperature of 73.0°C for the amplicons and 51.0°C for the snapback.

## 5. Conclusion and Recommendations

LSD is one of the most economically significant transboundary diseases in cattle. Despite the vaccination regime, the disease continues to appear almost every year on an outbreak level in Ethiopia, implying vaccination failure. The present study found that the disease was caused by LSDV because the collected samples were identified as LSDV using molecular techniques. Based on the findings of this study, the following recommendations are made to improve prevention and controlling strategies: further studies should be conducted targeting more regions and outbreaks, including full genome sequencing to check for genetic variations between field viruses and vaccine strains, and if possible, attenuation of field viruses for the development of a new vaccine based on the local isolate.

## Figures and Tables

**Table 1:** Outbreak investigation data in the study areas.

District	Cattle at risk	Diseased	Death	Morbidity rate (%)	Mortality rate (%)	Case fatality rate (%)
W/Jarso	9552	356	24	3.73	0.25	6.74
Amuru	7690	764	63	9.93	0.82	8.25
Total	17242	1120	87	6.50	0.50	7.77

## Data Availability

All data generated or analyzed during this study are available from the corresponding authors upon request.
